# Molecular Phylogeny of the *Myxobolus* and *Henneguya* Genera with Several New South American Species

**DOI:** 10.1371/journal.pone.0073713

**Published:** 2013-09-05

**Authors:** Mateus Maldonado Carriero, Edson A. Adriano, Márcia R. M. Silva, Paulo S. Ceccarelli, Antonio A. M. Maia

**Affiliations:** 1 Departamento de Medicina Veterinária, Faculdade de Zootecnia e Engenharia de Alimentos, Universidade de São Paulo, Pirassununga, São Paulo, Brazil; 2 Departamento de Ciências Biológicas, Universidade Federal de São Paulo, Diadema, São Paulo, Brazil; 3 Departamento de Biologia Animal, Instituto de Biologia, Universidade Estadual de Campinas, Campinas, São Paulo, Brazil; 4 Centro Nacional de Pesquisa e Conservação de Peixes Continentais, Instituto Chico Mendes de Conservação da Biodiversidade, Pirassununga, São Paulo, Brazil; Australian Museum, Australia

## Abstract

The present study consists of a detailed phylogenetic analysis of myxosporeans of the *Myxobolus* and *Henneguya* genera, including sequences from 12 *Myxobolus/Henneguya* species, parasites of South American pimelodids, bryconids and characids. Maximum likelihood and maximum parsimony analyses, based on 18 S rDNA gene sequences, showed that the strongest evolutionary signal is the phylogenetic affinity of the fish hosts, with clustering mainly occurring according to the order and/or family of the host. Of the 12 South American species studied here, six are newly described infecting fish from the Brazilian Pantanal wetland. *Henneguya maculosus* n. sp. and *Myxobolus flavus* n. sp. were found infecting both *Pseudoplatystoma corruscans* and *Pseudoplatystoma reticulatum*; *Myxobolus aureus* n. sp. and *Myxobolus pantanalis* n. sp. were observed parasitizing *Salminus brasiliensis* and *Myxobolus umidus* n. sp. and *Myxobolus piraputangae* n. sp. were detected infecting *Brycon hilarii*.

## Introduction

The phylum Myxozoa harbors a diverse group of metazoan parasites characterized by multicellular spores, with polar capsules containing an extrusible polar filament [1]. Parasites of this phylum have become increasingly important as new species are continually emerging as significant threats to the development of both farmed and natural environment fish [2].

The species have generally been described as infecting aquatic animals, especially fish, however many recent studies have reported the occurrence of myxozoans infecting mollusks [3], amphibians [4], [5], reptiles [6], [7], birds [8] and mammals [9]. As demonstrated firstly by Wolf and Markiw [10], it has been also proven that several other myxozoan species require an alternate invertebrate host (usually an annelid) to complete the life cycle [11]–[13].

Within myxozoans, 60 genera of the class Myxosporea and two of the class Malacosporea have been established. Among the myxosporeans, the genera *Myxobolus* Bütschli, 1882, and *Henneguya* Thélohan, 1892 are the most specious, and harbor species that have an important impact on their fish hosts [1], [2], [14], [15]. These two genera are considered separated groups mainly due the presence of caudal appendages in *Henneguya* spp. [16]. However, phylogenetic studies using 18 S rDNA sequence data do not support a phylogenetic separation between *Henneguya* and *Myxobolus* [17]–[19].

The traditional approach for taxonomic classification of Myxozoa is based on morphological characteristics, primarily of spores, but also of the plasmodia, as well as host and organ and/or tissue specificity. Nevertheless, spore morphology is the main basis of classification and identification, as the spores are relatively rigid and exhibit low intraspecific morphological variability [20]–[22]. However, molecular analyses have been an important tool in the study of these parasites since the late 1990s [8], [20], [23]. Molecular biological methods have now been employed in myxozoan research, using a number of different approaches, such as the morphological differentiation of similar species [24]–[26]; study of host and tissue specificity [27]; elucidation of life cycle [11], [23]; study of the phylogenetic relationships of the group [20], [28]–[32] and determination of the phylogenetic position of the myxosporeans inside the metazoans [33].

Some studies have attempted to establish the phylogenetic position of Myxozoa within Metazoa, but the classification of these parasites still remains uncertain, with evidence of the proximity of the myxozoans to both cnidarians and bilaterians, depending on the phylogenetic analysis used [33], [34]. However, in a recent study, Nesnidal et al. [35] constructed a phylogenetic tree based on a dataset including 128 genes, pointing out that these new genomic data unambiguously support the evolution of the parasitic Myxozoa from Cnidaria.

Other studies, such as those of Eszterbauer [36], Milanin et al. [30], Gleeson and Adlard [37], Hartigan et al. [5] and Adriano et al. [28], have investigated the phylogenetic relationships of the species within a particular genus. These studies have demonstrated that many factors influence species clustering, such as the phylogenetic proximity of the host, tissue tropism, geographic distribution and morphology characteristics.

The present study describes six new myxosporean species based on morphological and 18 S rDNA data and provides a detailed molecular phylogenetic analysis of the *Henneguya* and *Myxobolus* genera. This analysis includes the addition of sequences from 12 species of parasites of South American fishes and reveals the relationship of these species with parasites of fishes from other continents.

## Materials and Methods

All the fish handling was approved by the ethics committee for animal welfare of the Faculdade de Zootecnia e Engenharia de Alimentos of the Universidade de São Paulo (FZEA/USP), in accordance to the Brazilian legislation (Federal Law n° 11.794 from October 8th, 2008). The field samplings were carried out with permission of SISBIO (System Authorization and Information on Biodiversity) of the Ministry of Environment of Brazil (Authorization No. 15507-1).

Specimens of six fish species were caught in two Brazilian states. In the Brazilian Pantanal wetland (Pantanal National Park - 17°50′48′′S, 57°24′14′′W [distance variation of 3 km], municipality of Poconé, Mato Grosso State), fishes of three species from the family Pimelodidae were examined, including 21 specimens of *Pseudoplatystoma corruscans* Spix & Agassiz, 1829 (common name *pintado*), 12 of *Pseudoplatystoma reticulatum* Eigenmann & Eigenmann, 1889 Syn.: *Pseudoplatystoma fasciatum* Linnaeus, 1766 (common name *cachara*) and 4 specimens of *Zungaro jahu* Ihering, 1898 (common name *jaú*). In the family Bryconidae, 30 specimens of *Salminus brasiliensis* Cuvier, 1816 (common name *dourado*) and 27 of *Brycon hilarii* Valenciennes, 1850 (common name *piraputanga*) were examined. The examinations were performed from October 2008 to November 2009. Other examinations were performed in March 2010 in the Mogi Guaçu River, near the Cachoeira de Emas power plant, in Pirassununga, in the state of São Paulo (21°55′37″ S, 47°22′03″ W), where 4 specimens of *S. brasiliensis* were examined, and in a fish farm in Pirassununga (Centro Nacional de Pesquisa e Conservação de Peixes Continentais-CEPTA/ICMBio), where 5 specimens of the characid *Piaractus mesopotamicus* Holmberg, 1887 (common name *pacu*) were examined.

Immediately after capture, the fish were transported alive to the field laboratory where they were euthanized by benzocaine overdose and then measured and necropsied.

Organs or tissues infected with Myxozoa plasmodia were fixed in absolute ethanol for molecular analyses and in 10% buffered formalin for morphological analyses. Each sample was composed of several plasmodia grouped together according to phenotypic characteristics such as morphologic appearance, host species and tissue tropism.

In the laboratory, the plasmodia fixed in formalin were disrupted, and a small sample of the spores was transferred to a glass slide. A total of 30 spores of each sample were measured and photographed under a Leica DM 1000 light microscope equipped with Leica Application Suite version 1.6.0 image capture software. All measurements were performed according to the patterns established by Lom and Arthur [22]. These slides were then fixed with methanol and stained with Giemsa to be deposited in the collection of the Museum of Natural History, Institute of Biology, State University of Campinas (UNICAMP), state of São Paulo, Brazil.

DNA was extracted from the samples fixed in ethanol using the DNeasy® Blood & Tissue Kit (Qiagen) following the manufacturer’s instructions. The product was then quantified in a NanoDrop 2000 (Thermo Scientific, Wilmington, USA) spectrophotometer at 260 nm.

Polymerase chain reaction (PCR) was carried out, according to Adriano et al. [28] at a final volume of 25 µl using the primers MX5-MX3 (Table 1). These primers failed to amplify the samples of *Henneguya corruscans* Eiras et al., 2009 taken from *P. corruscans* and *P. reticulatum*, *Henneguya maculosus* n. sp. taken from *P. corruscans* and *Myxobolus pantanalis* n. sp. These samples were amplified with the primer pairs ERIB1-ACT1R and MYXGEN4f-ERIB10 (Table 1), which amplified two overlapping fragments of approximately 1,000 bp and 1,200 bp respectively of the 18 S rDNA gene. The other primers listed in Table 1 were used only in sequencing reactions.

**Table 1:** List of primers used in the amplification and sequencing of the 18 S rDNA gene.

Primers	Sequences	References
MX5 (forward)	5′-CTGCGGACGGCTCAGTAAATCAGT-3′	Andree et al. [20]
MX3 (reverse)	5′-CCAGGACATCTTAGGGCATCACAGA-3′	Andree et al. [20]
MC5 (forward)	5′-CCTGAGAAACGGCTACCACATCCA-3′	Molnár et al. [27]
MC3 (reverse)	5′-GATTAGCCTGACAGATCACTCCACGA-3′	Molnár et al. [27]
ERIB1(forward)	5′-ACCTGGTTGATCCTGCCAG-3′	Barta et al. [56]
ERIB10 (reverse)	5′-CTTCCGCAGGTTCACCTACGG-3′	Barta et al. [56]
MYXGEN4f (forward)	5′-GTGCCTTGAATAAATCAGAG-3′	Diamant et al. [57]
ACT1R (reverse)	5′-AATTTCACCTCTCGCTGCCA-3′	Hallett and Diamant [58]

The amplification reactions were conducted with 10–50 ng of genomic DNA, 2.5 µl of 1× Taq DNA Polymerase buffer (Invitrogen by Life Technologies, MD, USA), 0.75 µl of MgCl_2_ (1.5 mM), 0.5 µl of dNTPs (0.2 mM), 0.5 µl of each primer (0.2 µM), 0.25 µl of Taq DNA polymerase (2.5 U) (Invitrogen By Life Technologies, MD, USA) and MilliQ purified water. The PCR amplifications were performed in an AG 22331 Hamburg Thermocycler (Eppendorf, Hamburg, Germany). The PCR program consisted of 35 cycles of denaturation at 95°C for 60 s, annealing at 56°C for 60 s and extension at 72°C for 90 s, preceded by an initial denaturation at 95°C for 5 min and followed by a terminal extension at 72°C for 5 min.

The amplified products were analyzed via agarose gel electrophoresis, and their sizes were estimated by comparison with the 1 kb Plus DNA Ladder (Invitrogen by Life Technologies, CA, USA). The purified products were sequenced using the BigDye® Terminator v3.1 Cycle Sequencing kit (Applied Biosystems™) in an ABI 3730 DNA Analyzer (Applied Biosystems™ Inc., CA, USA) using the primers listed in Table 1.

The sequences were visualized, assembled and edited using BioEdit 7.1.3.0 [38] and, for each sequence, a standard nucleotide–nucleotide BLAST (blastn) search was conducted [39] to verify their similarity to other myxosporean sequences in GenBank.

Representative sequences of the 18 S rDNA gene of myxosporeans of the *Henneguya* and *Myxobolus* genera infecting almost every order of fish host available in GenBank were included in the analysis. Since the regions of the 18 S rDNA gene are highly conserved, sequences shorter than 1,000 nucleotides found in the GenBank were not included in the analysis in order to maintain as high a resolution of the resulting trees as possible and to avoid loss of information due to shortening of the aligned sequences and the appearance of too many alignment gaps, as indicated by Rosenberg and Kumar [40].

The sequences of *Kudoa thyrsites* and *Kudoa alliaria* were used as an outgroup. To avoid long-branch attraction effect due to distantly related outgroup sequences, two separated analyses were performed using both maximum likelihood and maximum parsimony methods. The first analysis was conducted with a set of aligned sequences containing outgroup sequences, and the second was performed with the same dataset, but excluding the outgroup. Since there were no substantial differences for topology and bootstrap values between the trees (with and without the outgroup), the tree containing the outgroup sequences was used as the base topology of the final tree.

A total of 114 sequences were aligned using ClustalW implemented in the program BioEdit 7.1.3.0 [38] with default settings. Manual adjustments were performed by eye to correct the alignment and remove ambiguous positions from the dataset, resulting in an alignment with a total of 2,270 characters, including alignment gaps. For *Myxobolus* spp. parasites of fish from the Eurasia Palearctic region, only sequences considered valid by Molnár [41] were used.

The best evolutionary model of nucleotide substitution using the Akaike Information Criterion (AIC) was determined with the program jModeltest 0.1 [42], which identified the general time reversible model (GTR+G) as the best fit. From the data, nucleotide frequencies (A = 0.2347, C = 0.1809, G = 0.2742, T = 0.3101) and the rates of the six different types of nucleotide substitution (AC = 1.2478, AG = 3.5282, AT = 1.5099, CG = 0.7026, CT = 4.1695, GT = 1.0000) were estimated. The alpha value of the gamma distribution parameter was 0.4200. These parameters were employed in maximum likelihood (ML) analysis, which was performed using PhyML 3.0 [43] software, with bootstrap confidence values calculated using 500 replicates.

A maximum parsimony (MP) analysis was performed using PAUP* 4.0b10 [44], with a starting tree obtained via stepwise addition and a heuristic search with 320,172 replicates, with a tree bisection–reconnection (TBR) branch swapping algorithm and random sequence addition. Clade supports were assessed via a bootstrap analysis of 1,000 replicates.

The resulting trees were visualized with TreeView 1.6.6 [45] and edited and annotated in Adobe Photoshop (Adobe Systems Inc., San Jose, CA, USA).

The sequences of the South American species were aligned to produce a pairwise similarity matrix using MEGA 5.0 [46], with possible gaps and/or missing data treated via complete deletion, to determine the relationships among them and to verifying the possible occurrence of the same myxosporean infecting different tissues or organs, geographic locations or host species.

### Nomenclatural Acts

The electronic edition of this article conforms to the requirements of the amended International Code of Zoological Nomenclature, and hence the new names contained herein are available under that Code from the electronic edition of this article. This published work and the nomenclatural acts it contains have been registered in ZooBank, the online registration system for the ICZN. The ZooBank LSIDs (Life Science Identifiers) can be resolved and the associated information viewed through any standard web browser by appending the LSID to the prefix “http://zoobank.org/”. The LSID for this publication is: urn:lsid:zoobank.org:pub:20E2B90B-801D-43E4-B6D0-CE0561FFF7EB. The electronic edition of this work was published in a journal with an ISSN, and has been archived and is available from the following digital repositories: PubMed Central, LOCKSS.

## Results

**Figure 1:**
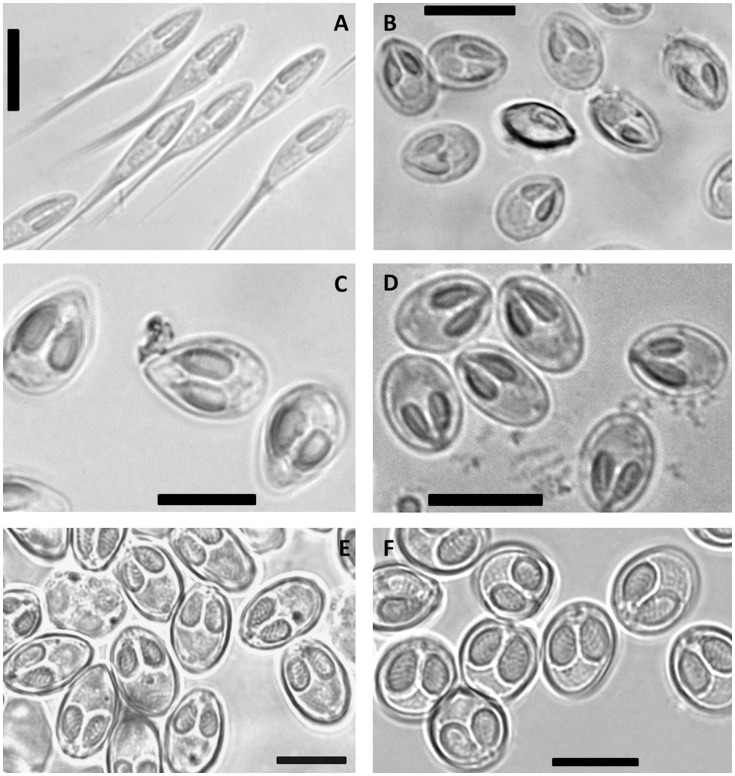
Photomicrographs of fresh spores of the novel myxosporean species. (A) *Henneguya maculosus* n. sp; (B) *Myxobolus flavus* n. sp.; (C) *Myxobolus aureus* n. sp.; (D) *Myxobolus pantanalis* n. sp.; (E) *Myxobolus umidus* n. sp.; (F) *Myxobolus piraputangae* n. sp. Scale bars = 10 µm.

**Figure 2:**
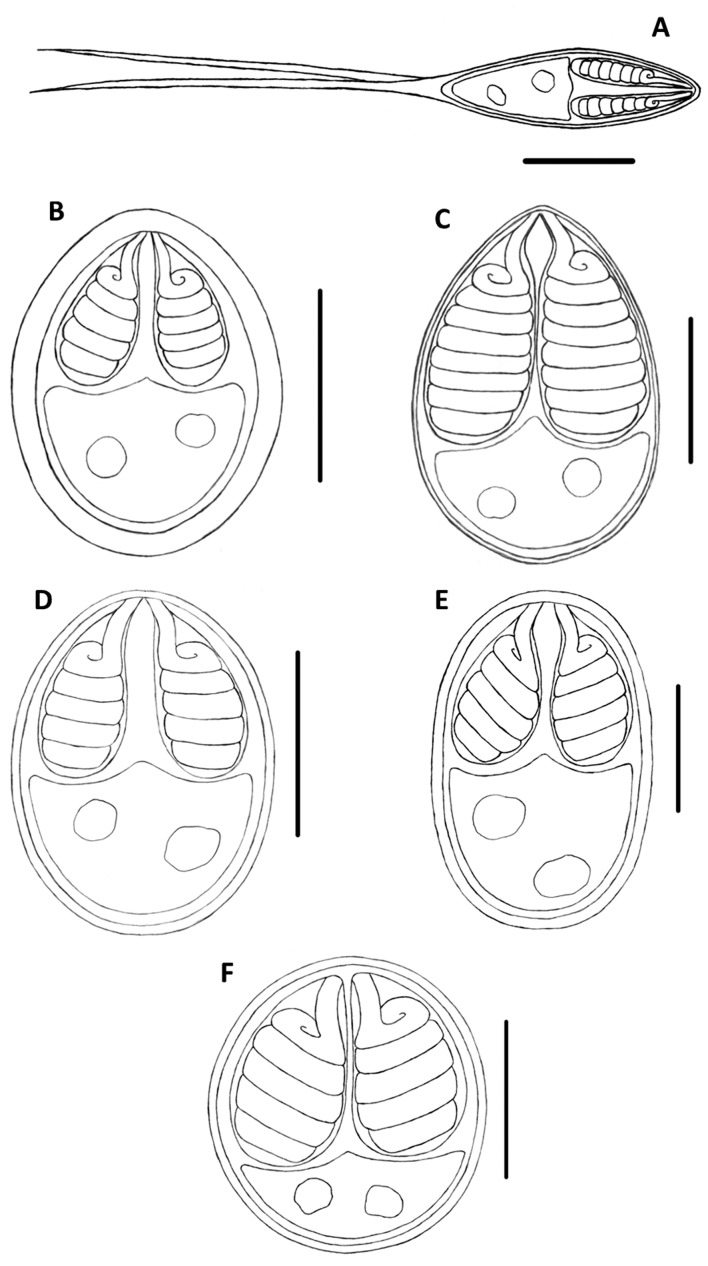
Schematic illustrations of the novel myxosporean species. (A) *Henneguya maculosus* n. sp.; (B) *Myxobolus flavus* n. sp.; (C) *Myxobolus aureus* n. sp.; (D) *Myxobolus pantanalis* n. sp.; (E) *Myxobolus umidus* n. sp.; (D) *Myxobolus piraputangae* n. sp. Scale bars = 5 µm.

Of the fishes examined in this study, the specimens of *P. corruscans* and *P. reticulatum* were infected by *H. corruscans*, *Henneguya multiplasmodialis* Adriano et al., 2012 and two undescribed myxosporeans, one *Henneguya* sp. that infected the gill filaments (Figs. 1A and 2A) and one *Myxobolus* sp. that infected the gill arch (Figs. 1B and 2B). *Henneguya eirasi* Naldoni et al., 2011 was found only in *P. reticulatum*. The specimens of *S. brasiliensis* from the Brazilian Pantanal wetland and Mogi Guaçu River were infected by *Myxobolus macroplasmodialis* Molnár et al., 1998, and those taken from the Pantanal wetland were infected with two undescribed *Myxobolus* spp., one parasitizing the liver (Figs. 1C and 2C) and another infecting the gill filaments (Figs. 1D and 2D). The specimens of *B. hilarii* were infected by *Myxobolus oliveirai* Milanin et al., 2010 and two undescribed *Myxobolus* spp., one of which infected the spleen (Figs. 1E and 2E), while the other parasitized the kidney (Figs. 1F and 2F). *Henneguya pellucida* Adriano et al., 2005 and *Myxobolus cordeiroi* Adriano et al., 2009 were found in *P. mesopotamicus* and *Z. jahu* respectively, with both infecting the serosa of the visceral cavity of their host.

Sequencing analysis produced 15 sequences from the 18 S rDNA gene of 12 different species parasites of South American freshwater fishes. Of these species, six consisted of still undescribed myxosporeans (Table 2; Figs. 1 and 2). The other six corresponded to described species: *M. macroplasmodialis* (one sequence obtained from parasites from the Brazilian Pantanal wetland and another from the Mogi Guaçu River), *H. pellucida*, *H. eirasi*, *H. multiplasmodialis, H. corruscans* and *M. cordeiroi*. For *M. macroplasmodialis* and *H. pellucida*, these were the first 18 S rDNA sequences obtained. For *H. corruscans*, the sequence produced herein was obtained from specimens found to infect *P. reticulatum*, which is a different host species than that from which it was originally described, and for *H. eirasi, H. multiplasmodialis* and *M. cordeiroi*, the new sequences were longer than the ones available in GenBank. The undescribed species, morphometric data, spores photomicrographs and drawings of the undescribed species are presented in the description provided below (Figs. 1 and 2; Table 2).

**Table 2:** Mean spore dimensions in µm ± standard deviation for the novel *Henneguya* and *Myxobolus* species from South America including their respective hosts and sites of infection.

Species	Spore length	Spore width	Spore thickness	TL	PCL	PCW	NFC	Infection site and host
*H. maculosus* n. sp.	13.7±0.6	4.1±0.2	3.0±0.2	17.5±1.0	5.6±0.5	1.6±0.2	6–7	Septated plasmodia in the gill filaments of *P. corruscans*
*H. maculosus* n. sp.	13.3±0.7	4.4±0.4	3.5±0.4	19.7±2.2	5.2±0.6	1.6±0.2	6–7	Septated plasmodia in the gill filaments of *P. reticulatum*
*M. flavus* n. sp.	9.2±0.2	6.5±0.3	4.2±0.2	–	4.5±0.2	1.6±0.1	4–5	Gill arch of *P. corruscans*
*M. flavus* n. sp.	9.3±0.3	6.6±0.3	4.0±0.2	–	4.5±0.2	1.8±0.1	4–5	Gill arch of *P. reticulatum*
*M. aureus* n. sp.	12.6±0.5	8.3±0.3	5.5±0.3	–	5.7±0.3	2.9±0.2	7–8	Liver of *S. brasiliensis*
*M. pantanalis* n. sp.	9.3±0.4	6.5±0.4	–	–	4.2±0.5	2.0±0.1	4–5	Gill filaments of *S. brasiliensis*
*M. umidus* n. sp.	13.5±0.7	7.8±0.4	7.7±0.1	–	5.1±0.4	2.7±0.3	4–5	Spleen of *B. hilarii*
*M. piraputangae* n. sp.	10.1±0.5	8.7±0.5	6.7±0.3	–	5.2±0.4	3.0±0.3	4–5	Kidney of *B. hilarii*

TL = tail length (only for *Henneguya maculosus* n. sp.); PCL = polar capsule length; PCW = polar capsule width; NFC = number of polar filament coils; dash = no data.

### Description of the New *Myxobolus* and *Henneguya* Species

#### Henneguya maculosus n. sp

urn:lsid:zoobank.org:act:68A6588A-510F-4BCD-829A-42B5FDE40236.

#### Description

White and elongated plasmodia, measuring 0.5 to 1.5 mm, found in the gill filaments of *P. corruscans* and *P. reticulatum*. The plasmodia exhibited spores in different stages of development. The mature spores were ellipsoidal from the frontal view, with the body measuring 13.7±0.6 µm in length, 4.1±0.2 µm in width, 3.0±0.2 µm in thickness and 17.5±1.0 µm in the caudal process. The polar capsules were elongated, filled the entire anterior half of the spore and were equal in size, measuring 5.6±0.5 µm in length and 1.6±0.2 in width. The polar filaments exhibited 6–7 turns. The measurements of the spores and polar capsules of the parasites found infecting *P. reticulatum* are presented in Table 2.

#### Type host

*Pseudoplatystoma corruscans* Spix & Agassiz, 1829;

#### Additional host

*Pseudoplatystoma reticulatum* Eigenmann & Eigenmann, 1889.

#### Prevalence

19% (4/21) in *P. corruscans* and 16.6% (2/12) in *P. reticulatum*.

#### Type locality

Pantanal National Park, state of Mato Grosso, Brazil.

#### Site of infection

Gill filaments.

#### Type material

Slides with stained spores (syntype) are deposited in the collection of the Museum of Natural History, Institute of Biology, State University of Campinas (UNICAMP), State of São Paulo, Brazil (accession number ZUEC – MYX 34).

#### Etymology

The specific name refers to the spots in the skin of the host, which is known in Portuguese as *pintado* (in English “spotted surubim”). In Latin *maculosus* = spotted (in English) = *pintado* (in Portuguese).

#### Remarks

**Figure 3:**
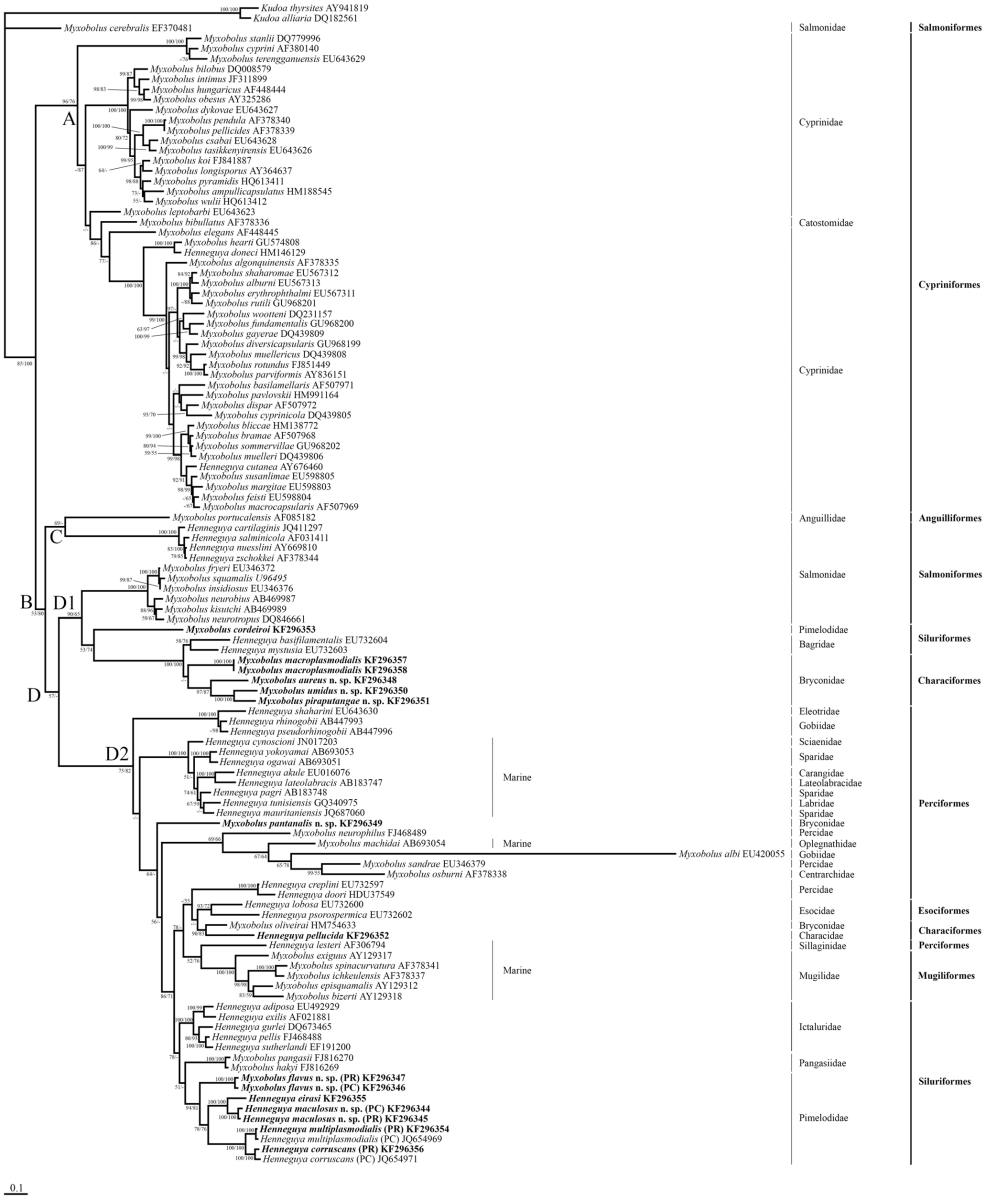
Maximum likelihood phylogenetic tree based on partial 18 S rDNA sequences. The bootstrap support values at branching points are listed as maximum likelihood/maximum parsimony. GenBank accession numbers are given following the name of the species. PW = Pantanal wetland; MG = Mogi Guaçu River; PC = *Pseudoplatystoma corruscans*; PR = *Pseudoplatystoma reticulatum*.

When compared with the other *Henneguya* spp. that have been described infecting *Pseudoplatystoma* fishes, *H. maculosus* n. sp. has spores with a morphology similar to that of *H. corruscans*. However, *H. maculosus* n. sp. has a longer caudal process, and the site of infection within the gills is different, as *H. maculosus* n. sp. forms plasmodia in the filaments, whereas *H. corruscans* forms round plasmodia on the lamellae. Apart from the length of the caudal process and the site of infection within the gills, the morphological features of the two species are very similar to each other, leading to inconclusive definitions regarding the separation of these species. However, combined with the differences mentioned above, molecular data (Table 3, Fig. 3) support the conclusion that these are distinct species.

**Table 3:** Similarity matrix for the 18 S rDNA sequences from all available *Henneguya* and *Myxobolus* species from South America adjusted for missing data (gaps).

Species	1	2	3	4	5	6	7	8	9	10	11	12	13	14	15	16	17	18
1. *H. maculosus* n. sp.(PC)		1.94	15.48	15.88	9.25	13.56	14.04	15.53	15.82	21.04	15.45	21.73	21.73	23.76	15.80	18.13	19.03	20.38
2. *H. maculosus* n. sp. (PR)	29		17.81	16.41	9.67	18.24	18.02	15.76	16.07	23.30	17.19	24.26	24.26	23.56	15.53	17.96	24.02	25.36
3. *H. multiplasmodialis* (PC)	240	265		0.91	15.92	7.03	7.02	15.79	16.03	24.76	18.52	23.86	23.86	23.34	17.42	18.50	27.04	25.95
4. *H. multiplasmodialis* (PR)	208	213	11		15.62	6.26	6.48	15.46	15.56	24.17	18.11	24.40	24.40	23.89	17.15	17.51	26.47	25.34
5. *H. eirasi*	111	116	187	184		16.40	17.13	15.93	16.00	24.31	17.70	27.10	27.10	24.52	16.12	17.68	23.46	25.07
6. *H. corruscans* (PC)	257	271	109	82	193		2.32	15.98	15.86	20.99	17.54	22.85	22.85	26.77	17.46	18.77	21.25	21.64
7. *H. corruscans* (PR)	265	268	109	85	202	44		15.17	15.50	21.21	17.68	22.17	22.17	25.94	18.09	18.75	21.48	21.92
8. *M. flavus* n. sp. (PC)	233	232	234	203	191	236	224		1.88	23.98	16.38	23.23	23.23	23.12	16.50	18.32	26.68	26.71
9. *M. flavus* n. sp. (PR)	239	240	239	203	192	235	230	28		24.37	16.87	24.18	24.18	23.19	16.33	19.21	26.54	26.74
10. *M. aureus* n. sp.	361	344	381	314	289	360	364	359	368		22.50	16.69	16.69	25.85	23.87	26.54	15.50	15.40
11. *M. pantanalis* n. sp.	290	253	287	237	209	328	331	244	253	389		22.36	22.36	25.98	15.96	15.83	22.92	23.66
12. *M. macroplasmodialis* (PW)	321	354	352	316	320	337	327	338	359	251	332		0.00	22.91	23.05	22.63	17.66	16.86
13. *M. macroplasmodialis* (MG)	321	354	352	316	320	337	327	338	359	251	332	0		22.91	23.05	22.63	17.66	16.86
14. *M. cordeiroi*	346	336	337	313	292	393	381	341	336	381	384	329	329		21.94	23.92	25.59	26.84
15. *M. oliveirai*	236	232	262	225	192	261	271	243	246	359	241	343	343	317		15.55	25.45	25.77
16. *H. pellucida*	280	268	279	229	212	285	285	277	291	414	244	336	336	354	235		26.33	27.37
17. *M. umidus* n. sp.	244	264	301	289	259	272	276	306	297	202	297	195	195	295	283	307		10.44
18. *M. pirautangae* n. sp.	258	279	288	276	276	275	279	305	299	199	304	185	185	310	286	320	135	

The lower triangular matrix shows the actual differences, while the upper triangular matrix shows the differences in terms of percentage of nucleotides. PC = *Pseudoplatystoma corruscans*; PR = *Pseudoplatystoma reticulatum*; PW = Pantanal wetland; MG = Mogi Guaçu River.

Sequences consisting of 1,930 and 1,496 nucleotides were produced from the samples of *H. maculosus* n. sp. infecting *P. corruscans* (GenBank accession number KF296344) and *P. reticulatum* (GenBank accession number KF296345) respectively. Between the sequences from the samples collected in the two hosts, there were variations in 29 nucleotides, which represent 1.94% of the comparable total. The South American species that presented the most similar sequence to *H. maculosus* n. sp. found in *P. corruscans* was *H. eirasi*, which exhibited 111 different nucleotides (9.25% of the total). The comparisons with the sequence from the sample found in *P. reticulatum* are displayed in Table 3.

#### Myxobolus flavus n. sp

urn:lsid:zoobank.org:act:EE0C4537-6A6B-4786-9B4A-4E651A2117EE.

#### Description

Yellow and spherical plasmodia measuring 1 to 5 mm and containing spores in different stages of development were found in the gill arch of. *P. corruscans* and *P. reticulatum*. The mature spores were oval shaped from the frontal view. The spores obtained from plasmodia collected from *P. corruscans* measured 9.2±0.2 µm in length and 6.5±0.3 µm in width. From the lateral view, the spores measured 4.2±0.2 µm, and the valves were symmetrical. The polar capsules were similar in size and measured 4.5±0.2 µm in length and 1.6±0.1 µm in width (Table 2). The polar filaments presented 4–5 turns. The measurements of the spores and polar capsules of the parasites found infecting *P. reticulatum* are displayed in Table 2.

#### Type host

Pseudoplatystoma corruscans Spix & Agassiz, 1829.

#### Additional host

*Pseudoplatystoma reticulatum* Eigenmann & Eigenmann, 1889.

#### Prevalence

38% (8/21) in *P. corruscans* and 33.3% (4/12) in *P. reticulatum*.

#### Type locality

Pantanal National Park, state of Mato Grosso, Brazil.

#### Site of infection

Gill arch.

#### Type material

Slides with stained spores (syntype) have been deposited in the collection of the Museum of Natural History, Institute of Biology, State University of Campinas (UNICAMP), State of São Paulo, Brazil (accession number ZUEC – MYX 39).

#### Etymology

The specific name refers to the yellow plasmodia formed by the parasite in the host, as in Latin, the word *flavus* = yellow.

#### Remarks

This is the first report of a species of the genus *Myxobolus* infecting fishes of the genus *Pseudoplatystoma*. The morphology of this parasite closely resembles that of *Myxobolus pantanalis* n. sp. (also described in this article). However, they are found in different host species and at different infection sites within the gills, with *M. pantanalis* n. sp. being found infecting the gill filaments of *S. brasiliensis* and *M. flavus* n. sp. obtained from the gill arch of *P. corruscans* and *P. reticulatum*. The molecular data show that these two myxosporeans do not share a close phylogenetic history. Compared to two other pimelodid-infecting *Myxobolus* species, *M. cordeiroi*, which infects *Z. jahu*, and *Myxobolus absonus* Cellere et al., 2002 infecting *Pimelodus maculatus* Lecépède, 1803 exhibit different morphological and morphometric characteristics in comparison to *M. flavus* n. sp.

Sequences with lengths of 1,520 and 1,530 bases of the 18 S rDNA gene were obtained from the hosts *P. corruscans* (GenBank accession number KF296346) and *P. reticulatum* (GenBank accession number KF296347) respectively. The difference between the sequences was 28 nucleotides, which represents 1.88% of the comparable total (Table 3). When compared to the other South American species, the smallest amount of interspecific differences was observed between *M. flavus* n. sp. infecting *P. corruscans* and *H. corruscans* infecting *P. reticulatum*, corresponding to 224 different nucleotides (15.17%). Comparisons with the sequence from the sample found in *P. reticulatum* are displayed in Table 3.

#### Myxobolus aureus n. sp

urn:lsid:zoobank.org:act:2EFA6C10-977B-4B56-B0A5-CC393833AEDB.

#### Description

Small white plasmodia, measuring approximately 0.4 mm in length found in the liver of *S. brasiliensis*. The mature spores were oval shaped from the frontal view and measured 12.6±0.5 µm in length and 8.3±0.3 µm in width. From the lateral view, the spores measured 5.5±0.3 µm, and the valves were smooth and symmetrical. The meeting of the two valves produced a tenuous sutural line. The two polar capsules showed the same size and measured 5.7±0.3 µm in length and 2.9±0.2 µm in width (Table 2). The polar filaments exhibited 7–8 turns.

#### Type host

*Salminus brasiliensis* Cuvier, 1816: Characiformes: Bryconidae.

#### Prevalence

30% (9/30).

#### Type locality

Pantanal National Park, state of Mato Grosso, Brazil.

#### Site of infection

Liver.

#### Type material

Slides with stained spores (syntype) have been deposited in the collection of the Museum of Natural History, Institute of Biology, State University of Campinas (UNICAMP), State of São Paulo, Brazil (accession number ZUEC – MYX 35).

#### Etymology

The specific name refers to the Portuguese common name of the host, *dourado* (in English, “golden”), arising from the golden color of *S. brasiliensis*. In Latin, *aureus* = golden.

#### Remarks

When compared to the other *Myxobolus* spp. found infecting *S. brasiliensis*, *M. aureus* n. sp. parasitizes the liver, whereas the previously described *Myxobolus salminus* Adriano et al., 2009 and *M. pantanalis* n. sp. (also described in this article) are both found infecting the gills, and *M. macroplasmodialis* is found in the visceral cavity. Regarding the morphology of *M. aureus* n. sp., its spores are similar to those of *M. salminus*, exhibiting a narrow anterior portion, but *M. aureus* n. sp. spores are longer and wider, with longer and wider polar capsules. Regarding the other *Myxobolus* spp. parasitizing characiforms, the morphology of *M. aureus* n. sp. does not resemble any of the species previously described.

A total of 1,757 bases of the 18 S rDNA gene of *M. aureus* n. sp. were sequenced (GenBank accession number KF296348). According to the similarity matrix (Table 3), the South American species that carries the most similar sequence to *M. aureus* n. sp. is *Myxobolus piraputangae* n. sp. (also described here), with 199 different nucleotides detected between them (15.40%) (Table 3).

#### Myxobolus pantanalis n. sp

urn:lsid:zoobank.org:act:A36FA08F-ACD6-4E9C-A415-E705044734D1.

#### Description

White, round-to-elongated plasmodia measuring approximately 0.2 to 1.2 mm in length and found in the posterior end (distal region) of the gill filaments of *S. brasiliensis*. The mature spores were oval shaped from the frontal view and measured 9.3±0.4 µm in length and 6.5±0.4 µm in width. The two polar capsules were elongated, pear shaped and equal in size and measured 4.2±0.5 µm in length and 2.0±0.1 µm in width (Table 2). The polar filaments exhibited 4–5 turns.

#### Type host

*Salminus brasiliensis* Cuvier, 1816: Characiformes: Bryconidae.

#### Prevalence

33.3% (10/30).

#### Type locality

Pantanal National Park, state of Mato Grosso, Brazil.

#### Site of infection

Gill filaments.

#### Type material

Slides with stained spores (syntype) have been deposited in the collection of the Museum of Natural History, Institute of Biology, State University of Campinas (UNICAMP), State of São Paulo, Brazil (accession number ZUEC – MYX 38).

#### Etymology

The specific name refers to the location in which the infected fishes were caught, the Brazilian Pantanal. In Latin, *pantanalis* = wetland.

#### Remarks

In *S. brasiliensis*, *M. salminus* was previously described as infecting the gills. However, the tissues infected by these two species are different, as *M. pantanalis* n. sp. is found in the surface of the filaments, and *M. salminus* is reported to infect the blood vessels of the gill filaments. These two species are also very different morphologically, as the spores of *M. pantanalis* n. sp. are ellipsoids with a round anterior end, while *M. salminus* exhibits a narrow anterior portion. Regarding the other *Myxobolus* spp. that infect *S. brasiliensis*, *M. aureus* n. sp. (described above) is found in the liver, and *M. macroplasmodialis* forms large plasmodia in the visceral cavity. All other *Myxobolus* spp. infecting characiforms differed from *M. pantanalis* n. sp. regarding morphological and morphometric aspects or other phenotypic features, such as the preferential site of infection and host species. As cited above, *M. pantanalis* n. sp. exhibited a similar morphology to *M. flavus* n. sp., however, they have different sites of infection and phylogenetically distant hosts.

A sequence consisting of 1,901 bases of the 18 S rDNA gene was produced from *M. pantanalis* n. sp. (GenBank accession number KF296349). According to the similarity matrix (Table 3), the smallest amount of interspecific differences was found with *H. maculosus* n. sp. parasitizing *P. corruscans*, corresponding to 290 different nucleotides (15.45%) (Table 3).

#### Myxobolus umidus n. sp

urn:lsid:zoobank.org:act:926BA436-7A00-4930-8614-C91CB6D57576.

#### Description

White and spherical plasmodia, measuring 0.4 to 0.8 mm, found in the spleen of *B. hilarii*. The mature spores were ellipsoid in shape from the frontal view and measured 13.5±0.7 µm in length and 7.8±0.4 µm in width. From the lateral view, the spores measured 7.7±0.1 µm, and the valves were symmetrical. The two polar capsules were oval shaped and of equal size, measuring 5.1±0.4 µm in length and 2.7±0.3 µm in width, and the polar filaments exhibited 4–5 turns, arranged obliquely with respect to the axis of the capsules (Table 2).

#### Type host

*Brycon hilarii* (Valenciennes, 1850*:* Characiformes: Bryconidae).

#### Prevalence

37% (10/27).

#### Type locality

Pantanal National Park, state of Mato Grosso, Brazil.

#### Site of infection

Spleen.

#### Type material

Slides with stained spores (syntype) have been deposited in the collection of the Museum of Natural History, Institute of Biology, State University of Campinas (UNICAMP), State of São Paulo, Brazil (accession number ZUEC – MYX 37).

#### Etymology

The specific name refers to the location in which the host is observed, the Brazilian Pantanal wetland, as in Latin, *umidus* = wet.

#### Remarks

When compared to the other *Myxobolus* spp. found infecting *B. hilarii*, *M. umidus* n. sp. differs from *M. oliveirai*, *Myxobolus brycon* Azevedo et al., 2011, and *Myxobolus piraputangae* n. sp. (also described in this article) in morphological and morphometric characteristics and other phenotypic features, such as the preferential site of infection, as this is the only species already described infecting the spleen of *B. hilarii*. Regarding species parasites of other characiform hosts, *Myxobolus serrasalmi* Walliker, 1969 exhibits spores with a similar size and is also found in the spleen of *Serrasalmus rhombeus* Linnaeus, 1766, but it has longer and wider polar capsules than *M. umidus* n. sp., as well as distinct host species.

A 1,307 base sequence of the 18 S rDNA gene was generated from *M. umidus* n. sp. (GenBank accession number KF296350). The South American species whose sequence presented the smallest amount of genetic differences compared to *M. umidus* n. sp. was *M. piraputangae* n. sp., with 135 different nucleotides (10.44%) (Table 3).

#### Myxobolus piraputangae n. sp

urn:lsid:zoobank.org:act:193C9F1F-4CAC-481B-A51C-06B77E8ED383.

#### Description

White and spherical plasmodia, measuring 0.5 to 1.0 mm, found in the kidney of *B. hilarii*. Mature spores were circular to slightly ellipsoidal in frontal view, measuring 10.1±0.5 µm in length and 8.7±0.5 µm in width. From the lateral view, the spores measured 6.7±0.3 µm, and the valves were symmetrical, with conspicuous pores for extrusion of the filaments in the anterior end of the spores. The two polar capsules were oval shaped and equal in size, measuring 5.2±0.4 µm in length and 3.0±0.3 µm in width and accounting for approximately two-thirds of the total spore length (Table 2). The polar filaments exhibited 4–5 turns arranged obliquely with respect to the axis of the capsules.

#### Type host

*Brycon hilarii* (Valenciennes, 1850*:* Characiformes: Bryconidae).

#### Prevalence

7.4% (2/27).

#### Type locality

Pantanal National Park, state of Mato Grosso, Brazil.

#### Site of infection

Kidney.

#### Type material

Slides with stained spores (syntype) have been deposited in the collection of the Museum of Natural History, Institute of Biology, State University of Campinas (UNICAMP), State of São Paulo, Brazil (accession number ZUEC – MYX 36).

#### Etymology

The specific name refers to the common name of the host, which is the *piraputanga* in Brazil.

#### Remarks

Compared to the other *Myxobolus* species infecting *B. hilarii*, *M. piraputangae* n. sp. exhibited different spore morphology, presenting circular spores, rather than pear shaped spores, as in *M. oliveirai* and *M. brycon*, or ellipsoidal spores, as in *M. umidus* n. sp. The dimensions of these species are also very different, as *M. oliveirai* and *M. umidus* n. sp. exhibit larger spores, while those of *M. brycon* are much smaller than the spores of *M. piraputangae* n. sp. Regarding the other *Myxobolus* spp. was found infecting other characiform fishes, *M. macroplasmodialis* was described infecting *S. brasiliensis* and *Myxobolus cunhai* Penido, 1927 was described infecting *Pygocentrus piraya* Cuvier, 1819, which have spores similar in size to those of *M. piraputangae* n. sp., however, they differ in other phenotypic aspects, such as spore morphology, the site of infection and the host species.

A 1,296 nucleotide sequence of the 18 S rDNA gene from *M. piraputangae* n. sp. was generated (GenBank accession number KF296351). The South American species presenting the sequence with the smallest genetic variation from that of *M. piraputangae* n. sp. was *M. umidus* n. sp., corresponding to 135 different nucleotides (10.44%) (Table 3).

### Phylogenetic Analyses

The partial 18 S rDNA gene sequences obtained from 15 samples in this study, plus three others from parasites of South American fishes, were compared with each other and with other myxozoan sequences from other geographic regions available in GenBank. BLAST searches demonstrated that these sequences exhibit a close relationship with those of other myxosporeans that have been previously sequenced, but they were not identical to any of them.

The similarity matrix of the 18 S rDNA gene sequences of the South American species showed that among the myxosporeans that infect more than one host, the intraspecific variation ranged from 0.91%, between the two isolates of *H. multiplasmodialis* found infecting *P. corruscans* and *P. reticulatum*, to 2.32%, between the samples of *H. corruscans* found infecting *P. corruscans* and *P. reticulatum* (Table 3). The *M. macroplasmodialis* sequences from *S. brasiliensis* sampled in the Brazilian Pantanal and the Mogi Guaçu River were identical. The smallest interspecific difference was observed between *H. multiplasmodialis* from *P. reticulatum* and *H. corruscans* from *P. corruscans*, which was 6.26%.

The phylogenetic analyses resulted in similar ML and MP topologies. Therefore, only the ML tree was used to represent the basic tree topology (Fig. 3), in which the clusters occurred mainly according to the phylogenetic affinities of the fish hosts. The tree was divided initially into two major clades, A and B. Clade A was formed by *Myxobolus/Henneguya* species associated exclusively with fishes belonging to the order Cypriniformes; clade B was further divided into two other clades (C and D); clade C consisted of one *Myxobolus* species parasite of anguiliforms and four *Henneguya* spp., parasites of salmoniforms; clade D was further divided to form two subclades (D1 and D2), both of *Myxobolus/Henneguya* species, which are parasites of different fish families. Subclade D1 harbored, in one branch, all the *Myxobolus* species parasites of salmoniforms, except *Myxobolus cerebralis* Hofer, 1903, which in our topology, was clustered outside the ingroup, much closer to the outgroup. In the other branch of the subclade D1, *M. cordeiroi,* a parasite of *Z. jahu*, a South American pimelodid, and *Henneguya basifilamentalis* Molnár et al., 2006 and *Henneguya mystusia* Sarkar, 1985, parasites of *Hemibagrus nemurus* Valenciennes, 1840, an Asian bagrid, were clustered. The branch D1 also harbored four *Myxobolus* species, parasites of South American characiforms of the bryconid family. The speciose subclade D2 harbored *Myxobolus/Henneguya* species parasites of marine perciforms and mugiliforms, two freshwater perciforms (*Henneguya creplini* Gurley, 1894 and *Henneguya doori* Guilford, 1963), plus *Henneguya lobosa* Cohn, 1895 and *Henneguya psorospermica* Thelohan, 1895 parasites of esociforms, and the South American *M. oliveirai* and *H. pellucida*, parasites of characiforms of the families Bryconidae and Characidae, respectively. *M. pantanalis* n. sp., another species that is parasite of a South American bryconid, appeared alone in a branch away from the other two groups of characiform parasites. Still in D2, five *Henneguya* spp., parasites of fishes of the family Ictaluridae, two *Myxobolus* spp., parasites of fishes of the pangasiid family, and five species (four *Henneguya* and one *Myxobolus*) that are parasites of fishes of the family Pimelodidae formed a large group composed of species that are parasites of siluriforms (Fig. 3).

## Discussion

In this report, based on analyses of the 18 S rDNA gene sequences and morphological features, six novel species of myxozoans are described, one of which belongs to the genus *Henneguya* and five to *Myxobolus*. Of these six new species, four *Myxobolus* spp. were found infecting bryconid hosts, with *M. aureus* n. sp. and *M. pantanalis* n. sp. being parasites of *S. brasiliensis* and *M. umidus* n. sp and *M. piraputangae* n. sp. parasitizing *B. hilarii*. In *S. brasiliensis* and *B. hilarii* four myxosporean species have been described previously, with *M. macroplasmodiaslis* and *M. salminus* parasitizing *S. brasiliensis* and *M. oliveirai* and *M. brycon* parasitizing *B. hilarii*. Both *M. flavus* n. sp. and *H. maculatus* n. sp. are described here as infecting two pimelodid species, *P. corruscans* and *P. reticulatum*. The observation of these myxosporeans in these two hosts expands the number of species described as infecting these pimelodids. The parasites previously reported in these fish species include *H. corruscans*, *H. eirasi* and *H. multiplasmodialis,* which were found infecting both hosts in the natural environment in Brazilian rivers, in addition to *Henneguya pseudoplatystoma* Naldoni et al., 2009, which was recorded infecting hybrid fish, resulting from a cross between *P. corruscans* and *P. reticulatum*, on fish farms in the São Paulo and Mato Grosso do Sul states, in Brazil.

The findings presented above reveal the notable fact that *P. corruscans* and *P. reticulatum*, which are species that are phylogenetically very close, can host the same myxosporean species. These results are in accordance with the host specificity criteria noted for *Henneguya* and *Myxobolus* species by Molnár [47] and Molnár et al. [48].

The similarity matrix showed that among the myxosporean species that are able to infect both *P. corruscans* and *P. reticulatum*, the genetic divergence between the samples obtained from the different hosts was only 0.91% for *H. multiplasmodialis*, 1.88% for *M. flavus* n. sp., 1.94% for *H. maculosus* n. sp. and 2.32% for *H. corruscans*. We consider these levels to reflect intraspecific divergences. On the other hand, the interspecific differences observed among the sequences of the morphologically distinct species varied from 6.26% to 27.37% (Table 3).

Might be expected that, because of the sampling method employed, in which several cysts from the same parasite species collected from each host species were grouped together to produce a satisfactory amount of DNA, we would detect intraspecific variations among samples from the same host. This might be observable on the sequencing electropherograms, but was not in fact detected. These results suggest that, although more specific studies regarding the population structure of this species with more suitable molecular markers are required, there exist different lineages of the same species infecting these two phylogenetically similar hosts, probably on a speciation process.

There is no exact value for determining whether particular differences in 18 S rDNA genes can be used to discriminate intra- or interspecific variation in members of the phylum Myxozoa [49]. Thus, this decision must be made for each individual case, always with the aid of other aspects of the biology and/or ecology of the organisms, such as their morphology, tissue and/or organ tropism, type of host and geographic area [49]. In taxonomic analyses of Myxozoa, Molnár et al. [50] observed a variation of 3.6% among samples of *Myxobolus dujardini* Thélohan, 1892, whereas in five other species, the intraspecific differences varied from 0.1% to 2.3%. In a study addressing isolates of *M. cerebralis* from different hosts and geographic locations, Andree et al. [20] observed a variation of only 0.8%. *Myxobolus fryeri* Ferguson et al., 2008 and *Myxobolus insidiosus* Wyatt & Pratt, 1963 were considered different species by Ferguson et al. [21] even though only 0.5% variation was detected between their 18 S rDNA sequences. Because the samples presented different infection sites and spores with significantly different lengths, the authors concluded that they were separate species. The same conclusion was made by Easy et al. [51], who stated that even though *Myxobolus intramusculi* Easy et al., 2005 and *Myxobolus procerus* Kudo, 1934 were both found in the muscles of *Percopsis omiscomaycus* Walbaum, 1792 and presented differences in only 2% of their 18 S rDNA sequences, they were considered two separate species, because *M. intramusculi* was found in intramuscular tissue, whereas from *M. procerus* was found infecting the adjacent connective tissue.

This is the first study investigating the phylogenetic relationships among several *Henneguya* spp. and *Myxobolus* spp. infecting South American fishes. In the ML and MP trees produced in this study, the characteristic presenting the most apparent phylogenetic signal was the relationship of the fish hosts, with clusters occurring mainly according to the order and family. This pattern can be clearly observed in almost all clades. Clade A was composed exclusively of *Myxobolus*/*Henneguya* species, parasites of fishes of the order Cypriniformes, indicating that this group has a distinct evolutionary origin from the other species analyzed. Following the tendency to group according to the order/family of the fish host, *Henneguya* spp. parasites of salmonids formed a subclade in clade C, while *Myxobolus* spp. parasites of salmonids formed a subclade in D1. However, *M. cerebralis* appears separated from the *Myxobolus* spp. parasites of salmonids, revealing that this group is not monophyletic. The South American species, parasites of characiform fishes also formed polyphyletic clusters, with species occurring in subclades D1 and D2. However, in subclade D1 there is a further subclade composed exclusively of four *Myxobolus* spp. parasites of the family Bryconidae. In clade D2, the characiform parasites *M. oliveirai* (from a bryconid host) and *H. pellucida* (from a characid host) clustered together, and *M. pantanalis* n. sp. clustered away from the other characiform parasites.

Regarding the *Myxobolus*/*Henneguya* species parasites of siluriforms, *M. cordeiroi*, parasite of a pimelodid and *H. basifilamentalis* and *H. mystusia,* parasites of an Asian bagrid, all clustered in a subclade of D1, while in a subclade of D2, all other species parasites of siluriforms (six *Henneguya* spp. of North American ictalurids, two *Myxobolus* spp. of an Asian pangasiid and four *Henneguya* spp. and one *Myxobolus* of South American pimelodids), formed a grouping composed exclusively of parasites of this host order. Despite the polyphyletic clustering observed among parasites of siluriforms, the large majority of the species clustered in a monophyletic subclade in D2, and even *M. cordeiroi*, *H. basifilamentalis* and *H. mystusia* clustered together in D1.

In general, even though there are some inconsistencies, as reported above, the tree produced in the present study shows a strong tendency of the *Myxobolus*/*Henneguya* species to form clusters based on the phylogenetic relationships of the fish hosts, even if these hosts are from different biogeographic regions, as has been previously reported by other authors [28], [32]. This suggests that the origins and radiations of these parasites are very ancient, perhaps as old as the hosts themselves, which, in the case of Osteichthyes, had an Early Silurian origin [52] and Mesozoic radiation [53].

The clustering of myxosporeans according to host phylogeny was also observed in a study by Gleeson et al. [54] which reported a clear separation among multivalvulid myxozoans of the genus *Kudoa*, parasites of elasmobranch and teleost hosts. However, this separation occurred at a higher taxonomic level of the hosts than that observed in this study for *Henneguya* and *Myxobolus*, since some *Kudoa* spp. have broader host specificity with infections across multiple families and even orders. Similar findings were reported by Gleeson and Adlard [37] who founded that *Chloromyxum* spp. parasites of elasmobranchs tend to cluster together despite having different geographic origins.

In clade D the occurrence of some clusters of lineages of myxozoans from a marine environment was observed, as were clusters with distribution according to the host order, with one clade composed of *Henneguya* spp. parasites of marine perciforms, and another formed by *Myxobolus* spp. parasites of mugiliforms. The only exception is the marine species *Myxobolus machidai* Li et al., 2012, which clustered with the estuarine *Myxobolus neurophilus* Guilford, 1963, *Myxobolus albi* Picon-Camacho et al., 2009 and *Myxobolus sandrae* Reuss, 1906, and the freshwater *Myxobolus osburni* Herrick, 1936.

Eszterbauer [36] suggested that the preference for a particular site of development plays an important role in determining the phylogenetic relationships among myxosporean species, and she also proposed that genetic separation based on tissue and/or organ tropism is a more ancient evolutionary characteristic than host specificity. However, the results reported by Eszterbauer [36] were based on data obtained from a study including only myxosporean species parasitizing cyprinid fishes. This does not mean that the site of infection is not a relevant evolutionary feature, but it appears that organ tropism has more evolutionary influence within clades that are defined by a stronger evolutionary signal, such as the order/family of the fish host. In accordance with Eszterbauer [36] and Fiala [18], our topology shows that tissue tropism has some phylogenetic influence in some places in the tree, especially when considering myxosporean species parasitizing hosts that are phylogenetically close. This effect can be observed in clade A, where the subclade formed by *Myxobolus diversicapsularis* Slukhai, 1984, *Myxobolus muellericus* Molnár et al., 2006, *Myxobolus rotundus* Nemeczek, 1911 and *Myxobolus parviformis* Kallert et al., 2005 is composed exclusively of *Myxobolus* species that parasitize the gills, while the subclade formed by *Myxobolus bliccae* Donec & Tozyyakova, 1984, *Myxobolus bramae* Reuss, 1906, *Myxobolus sommervillae* Molnár et al., 2010 and *Myxobolus muelleri* Bütschli, 1882 consists only of species that infect the blood vessels of the gills. A similar situation can be observed in clade C where, in the group of *Myxobolus* spp. parasites of salmoniforms, there is a clear separation between species infecting muscle (*M. fryeri, Myxobolus squamalis* Egusa et al., 1990 and *M. insidiosus*) and those infecting the central nervous system (*Myxobolus neurobius* Schuberg & Schröder, 1905, *Myxobolus kisutchi* Yasutake & Wood, 1957 and *Myxobolus neurotropus* Hogge et al., 2008). In all these cases, these myxosporean species clustered primarily by host affinity and in a second step, by organ/tissue tropism.

The classic taxonomic distinction between the *Myxobolus* and *Henneguya* genera is based on a single morphological character: the presence of the caudal process in *Henneguya* spp. and its absence in *Myxobolus* spp. [16]. Our phylogenetic analyses showed that species of the *Henneguya* and *Myxobolus* genera were grouped together, and this corroborates the results reported by several other authors in studies conducted in other regions of the world [17]–[19], [21]. This phenomenon can be observed in clade A, where *Henneguya doneci* Shulman, 1962 and *Henneguya cutanea* Dogiel & Petruschewsky, 1933 are found in a clade composed primarily of *Myxobolus* spp. The same situation occurred in clade D, where, as mentioned before, the parasites of bagrids *H. mystusia* and *H. basifilamentalis* clustered in a clade composed otherwise only of *Myxobolus* spp., as well as *M. oliveirai*, which clustered with *H. pellucida* and in the subclade composed of parasites of siluriforms, *Myxobolus hakyi* Baska et al., 2009, *Myxobolus pangasii* Molnár et al., 2006 and *M. flavus* n. sp. clustered in a predominantly *Henneguya* clade (Fig. 3). Thus, the findings of the present study of *Myxobolus/Henneguya* parasites of South American species, corroborate the idea that, based on analyses of ribosomal genes, there is no support for a phylogenetic separation of the *Henneguya* and *Myxobolus* genera. This absence of phylogenetic separation between these two genera led Kent et al. [17] to state that the caudal appendage of *Henneguya* spp. is not a valid feature for characterization of the genus. These authors also affirmed that the fact that species belonging the *Henneguya* and *Myxobolus* genera cluster together in groups with a monophyletic origin suggests that the genetic ability to develop spores with a caudal appendix is broadly distributed within the group, but due to still unknown reasons, only certain lineages express this character. A piece of evidence that provides support for this theory is the occurrence of some *Myxobolus* spp. that develop spores both with and without a *Henneguya*-like caudal prolongation within a single plasmodium, as reported for *Myxobolus turpisrotundus* Liu et al., 2010 [19], *Myxobolus bizerti* Bahri and Marques, 1996, *Myxobolus mulleri* Bahri, 1997 and *Myxobolus heterosporus* Baker, 1963 [55].

To date, phylogenetic studies of myxosporeans have been performed using species parasites from the Nearctic, Palearctic and Australian bioregions. In this study, for the first time, a consistent number of sequences of *Myxobolus*/*Henneguya* spp. parasites of fishes from the Neotropical region were included in a phylogenetic analysis with almost all of the valid sequences of species of these two genera available in GenBank. The results reveal that the strongest evolutionary signal for *Myxobolus/Henneguya* species is the phylogenetic affinity of the fish hosts, with clusters occurring mainly based on the order and/or family of the host. The origin of the Osteichthyes is estimated to the Early Silurian, around 427 million years ago [52], with Mesozoic radiation [53]. The observation that the phylogenetic affinity of the fish hosts is the strongest evolutionary signal in *Myxobolus/Henneguya* species suggests an ancient origin and radiation of this important and complex group of parasites.

## References

[1] Feist SW, Longshaw M (2006) Phylum Myxozoa. In: Woo PTK, editor. Fish diseases and disorders: Protozoan and Metazoan infections. 2^a^ ed. UK: CAB International. 230–296.

[2] Lom J, Dyková I (2006) Myxozoan genera: definition and notes on taxonomy, life-cycle terminology and pathogenic species. Folia Parasitol 53: 1–36. 16696428

[3] Yokoyama H, Masuda K (2001) *Kudoa* sp (Myxozoa) causing a post-mortem myoliquefaction of North-Pacific giant octopus *Paroctopus dofleini* (Cephalopoda : Octopodidae). B Eur Assoc Fish Pat 21: 266–268.

[4] Hartigan A, Fiala I, Dyková I, Jirků M, Okimoto B, et al. (2011) A Suspected parasite spill-back of two novel *Myxidium* spp. (Myxosporea) causing disease in australian endemic frogs found in the invasive cane toad. PLoS One 6. 10.1371/journal.pone.0018871 PMC3081827 21541340

[5] Hartigan A, Fiala I, Dyková I, Rose K, Phalen DN, et al (2012) New species of Myxosporea from frogs and resurrection of the genus *Cystodiscus* Lutz, 1889 for species with myxospores in gallbladders of amphibians. Parasitology 139: 478–496. 22260881 10.1017/S0031182011002149

[6] Eiras JC (2005) An overview on the myxosporean parasites in ampphibians and reptiles. Acta Parasitol 50: 267–275.

[7] Roberts JF, Whipps CM, Bartholomew JL, Schneider L, Jacobson ER (2008) *Myxidium scripta* n. sp identified in urinary and biliary tract of Louisiana-farmed red-eared slider turtles *Trachemys scripta elegans* . Dis Aquat Organ 80: 199–209. 18814545 10.3354/dao01912

[8] Bartholomew JL, Atkinson SD, Hallett SL, Lowenstine LJ, Garner MM, et al (2008) Myxozoan parasitism in waterfowl. Int J Parasitol 38: 1199–1207. 18342316 10.1016/j.ijpara.2008.01.008

[9] Prunescu CC, Prunescu P, Pucek Z, Lom J (2007) The first finding of myxosporean development from plasmodia to spores in terrestrial mammals: *Soricimyxum fegati* gen. et sp n. (Myxozoa) from *Sorex araneus* (Soricomorpha). Folia Parasit 54: 159–164. 10.14411/fp.2007.022 19245186

[10] Wolf K, Markiw ME (1984) Biology contravenes taxonomy in the Myxozoa - new discoveries show alternation of invertebrate and vertebrate hosts. Science 225: 1449–1452. 17770061 10.1126/science.225.4669.1449

[11] Atkinson SD, Bartholomew JL (2009) Alternate spore stages of *Myxobilatus gasterostei*, a myxosporean parasite of three-spined sticklebacks (*Gasterosteus aculeatus*) and oligochaetes (*Nais communis*). Parasitol Res 104: 1173–1181. 19107523 10.1007/s00436-008-1308-6

[12] Marton S, Eszterbauer E (2011) The development of *Myxobolus pavlovskii* (Myxozoa: Myxobolidae) includes an echinactinomyxon-type actinospore. Folia parasit 58: 157–163. 10.14411/fp.2011.015 21776895

[13] Székely C, Hallett SL, Atkinson SD, Molnár K (2009) Complete life cycle of *Myxobolus rotundus* (Myxosporea: Myxobolidae), a gill myxozoan of common bream *Abramis brama* . Dis Aquat Organ 85: 147–155. 19694174 10.3354/dao02068

[14] Eiras JC, Molnár K, Lu YS (2005) Synopsis of the species of *Myxobolus* Butschli, 1882 (Myxozoa : Myxosporea : Myxobolidae). Syst Parasitol 61: 1–46. 15928990 10.1007/s11230-004-6343-9

[15] Eiras JC, Adriano EA (2012) A checklist of new species of *Henneguya* Thélohan, 1892 (Myxozoa: Myxosporea, Myxobolidae) described between 2002 and 2012. Syst Parasitol 83: 95–104. 22983797 10.1007/s11230-012-9374-7

[16] Lom J, Dyková I (1992) Fine-structure of triactinomyxon early stages and sporogony - myxosporean and actinosporean features compared. J Protozool 39: 16–27.

[17] Kent ML, Andree KB, Bartholomew JL, El-Matbouli M, Desser SS, et al (2001) Recent advances in our knowledge of the Myxozoa. J Eukaryot Microbiol 48: 395–413. 11456316 10.1111/j.1550-7408.2001.tb00173.x

[18] Fiala I (2006) The phylogeny of Myxosporea (Myxozoa) based on small subunit ribosomal RNA gene analysis. Int J Parasitol 36: 1521–1534. 16904677 10.1016/j.ijpara.2006.06.016

[19] Liu Y, Whipps CM, Gu ZM, Zeng LB (2010) *Myxobolus turpisrotundus* (Myxosporea: Bivalvulida) spores with caudal appendages: investigating the validity of the genus *Henneguya* with morphological and molecular evidence. Parasitol Res 107: 699–706. 20512504 10.1007/s00436-010-1924-9

[20] Andree KB, Székely C, Molnár K, Gresoviac SJ, Hedrick RP (1999) Relationships among members of the genus *Myxobolus* (Myxozoa : Bilvalvidae) based on small subunit ribosomal DNA sequences. J Parasitol 85: 68–74. 10207366

[21] Ferguson JA, Atkinson SD, Whipps CM, Kent ML (2008) Molecular and morphological analysis of *Myxobolus* spp. of salmonid fishes with the description of a new *Myxobolus* species. J Parasitol 94: 1322–1334. 19127969 10.1645/GE-1606.1

[22] Lom J, Arthur JR (1989) A Guideline for the preparation of species descriptions in Myxosporea. J Fish Dis 12: 151–156.

[23] Xiao CX, Desser SS (2000) Cladistic analysis of myxozoan species with known alternating life-cycles. Syst Parasitol 46: 81–91. 10830830 10.1023/a:1006323207759

[24] Bahri S, Andree KB, Hedrick RP (2003) Morphological and phylogenetic studies of marine *Myxobolus* spp. from mullet in Ichkeul Lake, Tunisia. J Eukaryot Microbiol 50: 463–470. 14733438 10.1111/j.1550-7408.2003.tb00272.x

[25] Eszterbauer E, Benko M, Dan A, Molnár K (2001) Identification of fish-parasitic *Myxobolus* (Myxosporea) species using a combined PCR-RFLP method. Dis Aquat Organ 44: 35–39. 11253872 10.3354/dao044035

[26] Palenzuela O, Redondo MJ, Alvarez-Pellitero P (2002) Description of *Enteromyxum scophthalmi* gen. nov., sp. nov. (Myxozoa), an intestinal parasite of turbot (*Scophthalmus maximus* L.) using morphological and ribosomal RNA sequence data. Parasitology 124: 369–379. 12003061 10.1017/s0031182001001354

[27] Molnár K, Eszterbauer E, Székely C, Dan A, Harrach B (2002) Morphological and molecular biological studies on intramuscular *Myxobolus* spp. of cyprinid fish. J Fish Dis 25: 643–652.

[28] Adriano EA, Carriero MM, Maia AAM, Silva MRM, Naldoni J, et al (2012) Phylogenetic and host-parasite relationship analysis of *Henneguya multiplasmodialis* n. sp infecting *Pseudoplatystoma* spp. in Brazilian Pantanal wetland. Vet Parasitol 185: 110–120. 22051071 10.1016/j.vetpar.2011.10.008

[29] Fiala I, Bartošová P (2010) History of myxozoan character evolution on the basis of rDNA and EF-2 data. BMC Evol Biol 10: 228. 20667097 10.1186/1471-2148-10-228 PMC2927925

[30] Milanin T, Eiras JC, Arana S, Maia AA, Alves AL, et al (2010) Phylogeny, ultrastructure, histopathology and prevalence of *Myxobolus oliveirai* sp. nov., a parasite of *Brycon hilarii* (Characidae) in the Pantanal wetland, Brazil. Mem Inst Oswaldo Cruz 105: 762–769. 20944990 10.1590/s0074-02762010000600006

[31] Molnár K, Cech G, Székely C (2008) Infection of the heart of the common bream, *Abramis brama* (L.), with *Myxobolus* s.l. *dogieli* (Myxozoa, Myxobolidae). J Fish Dis 31: 613–620. 18700938 10.1111/j.1365-2761.2008.00904.x

[32] Naldoni J, Arana S, Maia AA, Silva MR, Carriero MM, et al (2011) Host-parasite-environment relationship, morphology and molecular analyses of *Henneguya eirasi* n. sp. parasite of two wild *Pseudoplatystoma* spp. in Pantanal Wetland, Brazil. Vet Parasitol 177: 247–255. 21237571 10.1016/j.vetpar.2010.12.008

[33] Evans NM, Holder MT, Barbeitos MS, Okamura B, Cartwright P (2010) The phylogenetic position of Myxozoa: exploring conflicting signals in phylogenomic and ribosomal data sets. Mol Biol Evol 27: 2733–2746. 20576761 10.1093/molbev/msq159

[34] Mallatt J, Craig CW, Yoder MJ (2012) Nearly complete rRNA genes from 371 Animalia: Updated structure-based alignment and detailed phylogenetic analysis. Mol Phylogenet Evol 64: 603–617. 22641172 10.1016/j.ympev.2012.05.016

[35] Nesnidal MP, Helmkampf M, Bruchhaus I, El-Matbouli M, Hausdorf B (2013) Agent of whirling disease meets orphan worm: phylogenomic analyses firmly place Myxozoa in Cnidaria. PLoS One 8. 10.1371/journal.pone.0054576 PMC3559788 23382916

[36] Eszterbauer E (2004) Genetic relationship among gill-infecting *Myxobolus* species (Myxosporea) of cyprinids: molecular evidence of importance of tissue-specificity. Dis Aquat Organ 58: 35–40. 15038449 10.3354/dao058035

[37] Gleeson RJ, Adlard RD (2012) Phylogenetic relationships amongst *Chloromyxum* Mingazzini, 1890 (Myxozoa: Myxosporea), and the description of six novel species from Australian elasmobranchs. Parasitol Int 61: 267–274. 22085584 10.1016/j.parint.2011.10.008

[38] Hall TA (1999) BioEdit: a user-friendly biological sequence alignment editor and analysis program for Windows 95/98/NT. Nucleic Acids Symp Ser 41: 95–98.

[39] Altschul SF, Madden TL, Schaffer AA, Zhang J, Zhang Z, et al (1997) Gapped BLAST and PSI-BLAST: a new generation of protein database search programs. Nucleic Acids Res 25: 3389–3402. 9254694 10.1093/nar/25.17.3389 PMC146917

[40] Rosenberg MS, Kumar S (2001) Incomplete taxon sampling is not a problem for phylogenetic inference. Proc Natl Acad Sci USA 98: 10751–10756. 11526218 10.1073/pnas.191248498 PMC58547

[41] Molnár K (2011) Remarks to the validity of Genbank sequences of *Myxobolus* spp. (Myxozoa, Myxosporidae) infecting Eurasian fishes. Acta Parasitol 56: 263–269.

[42] Posada D (2008) jModelTest: phylogenetic model averaging. Mol Biol Evol 25: 1253–1256. 18397919 10.1093/molbev/msn083

[43] Guindon S, Dufayard JF, Lefort V, Anisimova M, Hordijk W, et al (2010) New algorithms and methods to estimate maximum-likelihood phylogenies: assessing the performance of PhyML 3.0. Syst Biol 59: 307–321. 20525638 10.1093/sysbio/syq010

[44] Swofford DL (2003) PAUP*. Phylogenetic Analysis Using Parsimony (*and Other Methods). 4 ed. Sunderland, Massachusetts: Sinauer Associates.

[45] Page RD (1996) TreeView: an application to display phylogenetic trees on personal computers. Comput Appl Biosci 12: 357–358. 8902363 10.1093/bioinformatics/12.4.357

[46] Tamura K, Peterson D, Peterson N, Stecher G, Nei M, et al (2011) MEGA5: Molecular evolutionary genetics analysis using maximum likelihood, evolutionary distance, and maximum parsimony methods. Mol Biol Evol 28: 2731–2739. 21546353 10.1093/molbev/msr121 PMC3203626

[47] Molnár K (1998) Taxonomic problems, seasonality and histopathology of *Henneguya creplini* (Myxosporea) infection of the pikeperch *Stizostedion lucioperca* in Lake Balaton. Folia Parasitol 45: 261–269. 9868790

[48] Molnár K, Ranzani-Paiva MJ, Eiras JC, Rodrigues EL (1998) *Myxobolus macroplasmodialis* sp. n. (Myxozoa : Myxosporea), a parasite of the abdominal cavity of the characid teleost, *Salminus maxillosus*, in Brazil. Acta Protozool 37: 241–245.

[49] Gunter NL, Adlard RD (2009) Seven new species of *Ceratomyxa* Thélohan, 1892 (Myxozoa) from the gall-bladders of serranid fishes from the Great Barrier Reef, Australia. Syst Parasitol 73: 1–11. 19337855 10.1007/s11230-008-9162-6

[50] Molnár K, Marton S, Eszterbauer E, Székely C (2006) Comparative morphological and molecular studies on *Myxobolus* spp. infecting chub from the river Danube, Hungary, and description of *M. muellericus* sp. n. Dis Aquat Organ 73: 49–61. 17240752 10.3354/dao073049

[51] Easy RH, Johnson SC, Cone DK (2005) Morphological and molecular comparison of *Myxobolus procerus* (Kudo, 1934) and *M. intramusculi* n. sp. (Myxozoa) parasitising muscles of the trout-perch *Percopsis omiscomaycus* . Syst Parasitol 61: 115–122. 15980965 10.1007/s11230-005-3135-9

[52] Broughton RE, Betancur RR, Li C, Arratia G, Orti G (2013) Multi-locus phylogenetic analysis reveals the pattern and tempo of bony fish evolution. PLoS Curr 5. 10.1371/currents.tol.2ca8041495ffafd0c92756e75247483e PMC3682800 23788273

[53] Nakatani M, Miya M, Mabuchi K, Saitoh K, Nishida M (2011) Evolutionary history of Otophysi (Teleostei), a major clade of the modern freshwater fishes: Pangaean origin and Mesozoic radiation. BMC Evol Biol 11: 177. 21693066 10.1186/1471-2148-11-177 PMC3141434

[54] Gleeson RJ, Bennett MB, Adlard RD (2010) First taxonomic description of multivalvulidan myxosporean parasites from elasmobranchs: *Kudoa hemiscylli* n.sp. and *Kudoa carcharhini* n.sp. (Myxosporea: Multivalvulidae). Parasitology 137: 1885–1898. 20619061 10.1017/S0031182010000855

[55] El-Mansy A (2005) Revision of *Myxobolus heterosporus* Baker, 1963 (syn. *Myxosoma heterospora*) (Myxozoa : Myxosporea) in African records. Dis Aquat Organ 63: 205–214. 15819436 10.3354/dao063205

[56] Barta JR, Martin DS, Liberator PA, Dashkevicz M, Anderson JW, et al (1997) Phylogenetic relationships among eight *Eimeria* species infecting domestic fowl inferred using complete small subunit ribosomal DNA sequences. J Parasitol 83: 262–271. 9105308

[57] Diamant A, Whipps CM, Kent ML (2004) A new species of *Sphaeromyxa* (Myxosporea : Sphaeromyxina : Sphaeromyxidae) in devil firefish, *Pterois miles* (Scorpaenidae), from the northern Red Sea: Morphology, ultrastructure, and phylogeny. J Parasitol 90: 1434–1442. 15715240 10.1645/GE-336R

[58] Hallett SL, Diamant A (2001) Ultrastructure and small-subunit ribosomal DNA sequence of *Henneguya lesteri* n. sp (Myxosporea), a parasite of sand whiting *Sillago analis* (Sillaginidae) from the coast of Queensland, Australia. Dis Aquat Organ 46: 197–212. 11710554 10.3354/dao046197

